# Test–retest reliability for performance-based outcome measures among individuals with arthrogryposis multiplex congenita

**DOI:** 10.1186/s12891-022-05070-w

**Published:** 2022-02-05

**Authors:** Jaclyn Megan Sions, Maureen Donohoe, Emma Haldane Beisheim-Ryan, Ryan Todd Pohlig, Tracy Michele Shank, Louise Reid Nichols

**Affiliations:** 1 Department of Physical Therapy, University of Delaware, 540 South College Ave., Suite 210JJ, Newark, Delaware 19713 USA; 2 Therapeutic and Rehabilitative Services Department, Nemours Alfred I. duPont Hospital for Children, 1600 Rockland Road, Wilmington, Delaware 19803 USA; 3 Department of Physical Therapy, University of Delaware, 540 South College Ave., Suite 144A, Newark, Delaware 19713 USA; 4 VA Eastern Colorado Geriatric Research, Education, and Clinical Center (GRECC), VA Eastern Colorado Health Care System, 1700 North Wheeling Street, Aurora, CO 80045-7211 USA; 5 Biostatistics Core, University of Delaware, 102B STAR Tower, Newark, Delaware 19713 USA; 6 Orthopedics Department, Nemours Alfred I. duPont Hospital for Children, 1600 Rockland Road, Wilmington, Delaware 19803 USA

**Keywords:** Arthrogryposis, Clubfoot, Gait, Postural balance, Walking speed

## Abstract

**Background:**

Most individuals with arthrogryposis multiplex congenita, a rare condition characterized by joint contractures in ≥ 2 body regions, have foot and ankle involvement leading to compromised gait and balance. The purpose of this study was to establish between-days, test–retest reliability for performance-based outcome measures evaluating gait and balance, i.e., the 10-m Walk Test, Figure-of-8 Walk Test, 360-degree Turn Test, and modified Four Square Step Test, among adolescents and adults with arthrogryposis multiplex congenita.

**Methods:**

This reliability study included ambulatory participants, aged 10 to 50 years, with a medical diagnosis of arthrogryposis multiplex congenita. Participants completed performance-based measures, in a randomized order, on two separate occasions. Intraclass correlation coefficients with 95% confidence intervals and minimal detectable changes at the 90% and 95% confidence level were calculated.

**Results:**

Participants included 38 community-ambulators with a median of 13 out of 14 upper and lower joint regions affected. Intraclass correlation coefficient point estimates and 95% confidence intervals ranged from .85-.97 and .70-.98, respectively. Minimal detectable changes were 10 to 39% of sample means and were largest for the modified Four Square Step Test.

**Conclusions:**

Among individuals with arthrogryposis, gait speed per the 10-m Walk Test, as well as non-linear walking and dynamic balance assessment per the Figure-of-8 Walk and 360 Degree Turn Tests, have adequate test–retest reliability enabling evaluation of individual patient changes. Changes in groups of ambulatory individuals with arthrogryposis multiplex congenita may be reliably evaluated with all of the studied outcome measures.

## Background

Arthrogryposis multiplex congenita (AMC) is a condition occurring in about 1 in 5,000–10,000 live births characterized by non-progressive joint contractures in 2 or more body regions [1–3]. Clinical presentations are variable, resulting in considerable heterogeneity of individuals with AMC in terms of mobility status, cognitive status, and limitations with activities-of-daily living [1, 4, 5]. Early clinical courses include extensive serial casting, orthotics management, rehabilitation, and often, orthopedic surgeries [6–9]. Care of the lower-extremities focuses on optimizing independence through maximizing ambulatory potential. After optimization, there is a need to monitor for functional degradation, particularly as children transition from adolescence into early adulthood, when they may no longer have access to AMC-provider specialists as they age-out of pediatric healthcare facilities [10]. Longitudinal monitoring requires quick and reliable outcome measures with known minimal detectable changes (MDCs) that allow assessment of whether ‘true change’ exceeding measurement error has occurred [11]. 

Performance-based outcome measures evaluate an individual’s capacity under a given set of conditions, complementing patient-reported outcomes, which evaluate an individual’s perceived ability [12]. While self-report measures are discriminative when evaluating if individuals are able or unable to perform a given task, performance-based measures may further stratify levels of physical functioning [12]. Assuming standardized testing procedures, an additional inherent benefit to timed performance-based measures is objectivity. Objectivity may be critical when justifying care, including surgical procedures, orthotics, assistive technology, and rehabilitation, particularly when there is a potential conflict of interest for the examiner and/or patient.

Whereas performance-based functional outcomes, such as the 10-m Walk Test (10mWT), are staples in adult rehabilitation, use in pediatric orthopedics is less common, especially among patients with congenital conditions, including AMC [7]. If non-condition-specific performance-based outcome measures used in other patient populations are established as psychometrically-sound among children and adults with AMC, practitioners may be able to easily track function from adolescence through adulthood, without the need for novel measures, overcoming a primary barrier to adoption of new outcome measures-lack of practitioner familiarity [13]. Further, use of outcome measures that are not condition-specific allows for comparison to normative data, as well as comparison across patient samples; such comparisons can assist with prioritization of patient subgroups most in need of limited resources due to greater risk for poor outcomes.

Among adolescents and young adults with AMC, lower-extremity involvement, especially of the foot and ankle, occurs in 80–90% of individuals [14]. Consequently, gait and balance impairments are ubiquitous. In community-dwelling older adults, reduced gait speed is predictive of adverse health outcomes, such as disability, institutionalization, and mortality [15]. Among pediatric populations, reduced gait speed is associated with worse disease severity and greater disability, [16, 17] and in some populations like Hereditary Motor Sensory Neuropathy, gait speed is a predictor of subsequent functional decline [18]. Given increased challenges to gait stability and symmetry with curved path walking, the use of straight-path walking alone is discouraged [19]. Thus, the purpose of this study was to establish between-days, test–retest reliability and minimal detectable changes for four outcome measures of gait and balance among adolescents and adults with AMC. The four measures were the 10mWT, which evaluates straight-path walking, and the Figure-of-8 Walk Test (F8WT), 360-degree Turn Test (360TT), and modified Four Square Step Test (mFSST), which evaluate non-linear walking.

## Methods

### Study design and participants

**Fig. 1:**
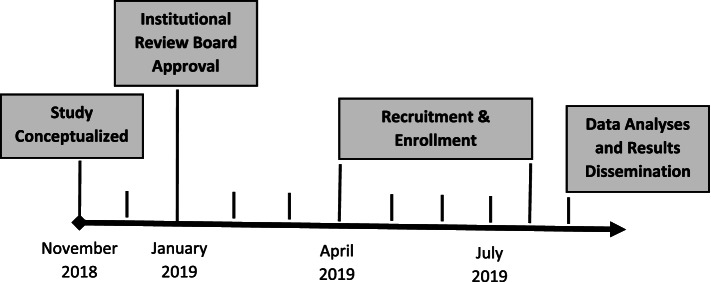
Study Timeline

This test–retest reliability study recruited participants, aged 10 to 50 years, with a medical diagnosis of AMC who were ambulatory and able to walk at least 30 m with or without an assistive device. Recruitment occurred through verbal recruitment at community events and print advertisements from April to July of 2019. Exclusion criteria included cognitive-impairment precluding assent/consent, spine or lower-limb surgery in the past 6 months, a history of lower-limb amputation, current dizziness, an acute illness, or a progressive neuromuscular disease. The study was conceptualized in November of 2018, approved by the University of Delaware Institutional Review Board for Human Subjects Research (project number: 1354682; initial approval date: 1/16/2019) and conformed to the World Medical Association’s Helsinki Declaration; written informed consent/assent and parental permission (as applicable based on age) was obtained for all participants. See Fig. 1 for the study timeline.

### Data collection

Data collections occurred at a University of Delaware clinical research laboratory in Newark, Delaware, and at a Hilton hotel conference space in Norfolk, Virginia. After informed consent, trained research staff conducted standardized interviews for participant characterization; parental input was encouraged for medical history recall among adolescent participants. Participants independently completed the Gillette Functional Assessment Questionnaire to characterize their mobility status, where scores range from 1 = “unable to take steps'' to 10 = “walks, runs, climbs on level and uneven terrain without difficulty or assistance” [20]. For further participant characterization, average pain intensity rating over the past 7 days was obtained with the Patient-Reported Outcomes Measurement Information System, where 0 = “no pain” and 10 = “worst imaginable pain” [21]. Participants then completed performance-based outcome measures in a randomized order, and returned 1–10 days later for repeat performance-based testing.

### Performance-based outcome measures

#### 10mWT

**Fig. 2:**
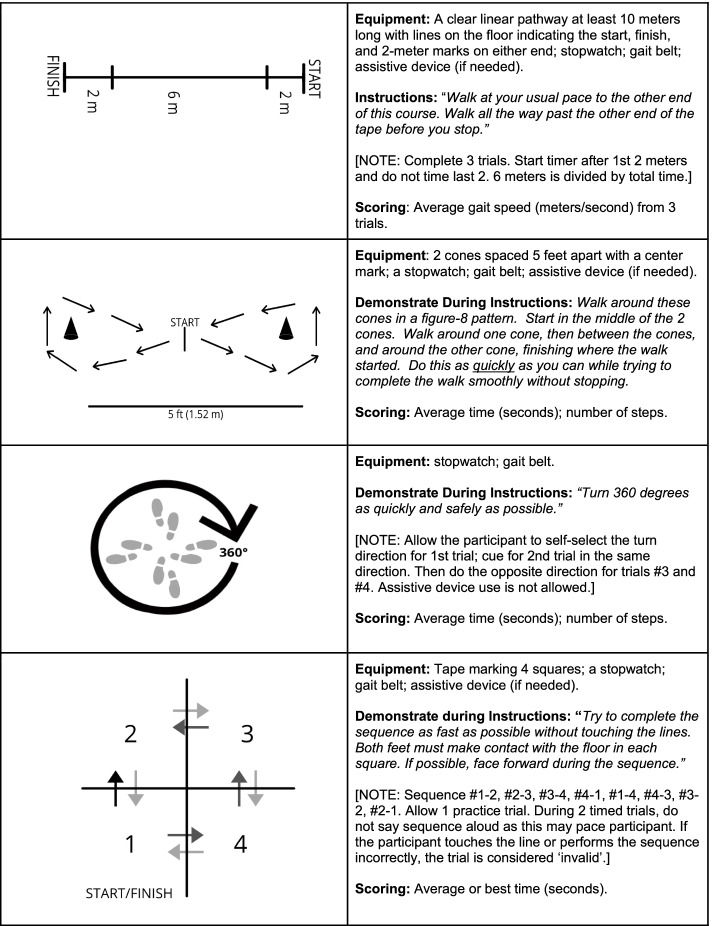
Standardized Procedures for Performance-Based Outcome Measures

‘Usual’ gait speed was obtained over the middle 6 m of a 10-m course, allowing 2 m for acceleration and deceleration at either end (Fig. 2) [22]. For some children, adolescent, and adult populations, such as those with neurological conditions and hip dysplasia, between-days test–retest reliability for the 10mWT has been reported [23, 24]. The 10mWT, using ≥ 2 trials, is included in a core set of rehabilitation outcome measures recommended for adults with neurological conditions based on its established psychometric properties and clinical utility [22]. Gait speed was determined from a three-trial average based on prior test–retest reliability research [25].

#### F8WT

The F8WT, which times curved-path walking around two cones arranged in a figure-of-8 (Fig. 2), is a valid measure of walking skill, and provides complimentary information to gait speed [26]. F8WT can differentiate between adults with lower-limb pathology with varying functional mobility levels [27]. Among adults post-stroke and -total knee arthroplasty, test–retest reliability for time and number of steps, where a greater number of steps indicates poorer performance, has been reported [26, 28–30].

#### 360TT

During community ambulation, up to 50% of steps may include turning, [31] and impaired turning has been associated with recurrent falls [32]. Rehabilitation balance batteries, including the Pediatric Balance Scale, Berg Balance Scale, and Tinetti Performance-Oriented Mobility Assessment, have a 360-degree turn task, but administration time required to complete such batteries may reduce clinical adoption [33]. Therefore, in this study, four 360-degree turns [two to the left; two to the right; [34] Fig. 2] were completed and average time for completion and number of steps were determined. More steps to complete the turn indicates poorer performance. Test–retest reliability for 360TT, in isolation, has been reported in adult patient populations, such as those with Multiple Sclerosis, Parkinson’s disease, and post-stroke [34–37].

#### mFSST

In various pediatric and adult populations, Four Square Step Test (FSST) test–retest reliability has been reported, as has concurrent, construct, and predictive validity for falls among adults [23, 38–40]. With the FSST, individuals complete multi-directional stepping (i.e., forwards, lateral, and backwards) over canes arranged in a ‘ + ’ in a specified sequence [38]. Requirement of a specified sequence for a valid trial increases cognitive-load, but the requirement for foot clearance alongside correct sequencing can result in ‘invalid’ trials, contributing to the FSST’s known floor effect [38]. Thus, the mFSST, which substitutes taped lines for canes, was used (Fig. 2) [27, 39]. Aligning with prior research, [39] reliability for ‘average’ and ‘best’ performance over two trials, was determined.

### Data Analyses

IBM SPSS Statistics 26 (Armonk, NY, USA) was used for all analyses. Descriptive statistics were determined. Wilcoxon Signed Ranks Test was used to evaluate intra-individual differences in pain intensity between testing sessions (p ≤ 0.050). Intraclass correlation coefficients (ICCs) were used to evaluate between-days, test–retest reliability for performance-based outcome measures using two-way mixed effects, absolute agreement (ICC_3,1 or 3,k_ models) [41, 42]. Given the nonparametric distribution of performance-based data, Bland–Altman plots were evaluated to ensure ICC analyses were appropriate. ICCs > 0.90 may be considered excellent [41] and desirable for evaluating individual patient changes, [43] while ICCs > 0.70 may be adequate for evaluating group changes [44, 45]. Standard errors of measurements (SEMs) were calculated. The decision to use MDC at the 90% (MDC_90_) or 95% confidence level (MDC_95_) may be determinant on the intervention. For example, meeting or exceeding MDC_90_ may be appropriate for evaluating success of conservative interventions, such as rehabilitation, but when evaluating success of surgical interventions with greater inherent risks, MDC_95_ may be more appropriate; [46] thus, both MDC_90_ and MDC_95_ were determined.

## Results

Of the 63 interested individuals with AMC screened for study participation, 12 were ineligible due to an inability to walk at least 30 m. Fifty-one enrolled participants completed the first onsite data collection, which was part of a larger cross-sectional research project. Of these 51 participants, 38 were available and agreed to participate in an optional second onsite data collection within 1–10 days. Thus, 38 participants were included in this between-days, test–retest reliability study, exceeding the recommended sample size of at least 30 participants for reliability studies in rehabilitation [47].

The sample was largely female, Caucasian, and reported no known genetic cause for their AMC (Table 1). Nearly 50% of the sample had spinal involvement; the median number of affected upper-limb regions was 8 out of 8, and the median number of affected lower-limb regions was 5 out of 6. Home assistive device use was rare, but 29% of the sample reported community assistive device use. The median number of lower-limb orthopedic surgeries was 4, with spinal surgeries and upper-limb surgeries reported much less frequently. Most participants were ambulatory outside the home for community distances (i.e., GFAQ ≥ 8), and had mild-to-moderate pain over the course of last 7 days. Pain was not significantly different between testing sessions 1 and 2 (*p* = 0.368), with approximately two-thirds of the sample reporting 0/10 pain at the time of performance-based testing.

**Table 1:** Participant Characteristics (*n* = 38)

**Age, y**^a^	20 (14, 33)
**Sex, female**	27 (71.1)
**Ethnicity, Non-Hispanic/Latino**	34 (89.5)
**Race, white/Caucasian**	31 (81.5)
**Height, m**^a^	1.56 (1.45, 1.63)
**Weight, kg**^a×^	53.1 (40.8, 62.4)
**Known Genetic Cause of AMC**	4 (10.5)
**Spinal Involvement** ×	18 (48.6)
**Upper Limb Regions, 0-8**^a^	8 (4, 8)
**Lower Limb Regions, 0-6**^a^	5 (3, 6)
**ASSISTIVE TECHNOLOGY**	
**Lower Limb Orthotics Use**	22 (57.9)
**Home Assistive Device Use**	1 (2.6)
Cane	1 (2.6)
**Community Assistive Device Use**	11 (28.9)
Cane	3 (7.9)
Loftstrand Crutches	2 (5.3)
Rollator Walker	1 (2.6)
Wheelchair	5 (13.2)
**Spinal**	6 (15.8)
**Number of Upper Limb**^a**↑**^	0 (0, 3)
**Number of Lower Limb**^a**↓**^	4 (2, 7)
**GFAQ**^a^	9 (8, 9)
**7-day Average Pain Rating, 0-10**^a^	3 (1, 4)

^a^Data presented as median (25^th^, 75^th^ percentile) rather than n (% of sample)

^X^*n* = 1 participant with missing data

^β^*n* = 2 participants with missing data

^↑^upper-limb regions included right and left shoulder, elbows, wrists, and hands

^↓^lower-limb regions included right and left hips, knees, and ankle/foot complex

*Abbreviations*: *GFAQ* Gillette Functional Assessment Questionnaire, *y* years

Bland–Altman plots for performance-based measures indicated ICC analyses were appropriate; however, differences between timepoints were significantly different from 0 for F8WT, average mFSST, and best mFSST scores (t = 2.061–3.783, *p* = 0.001-0.047). It was not unexpected for a practice effect to occur with repeated trials of novel tasks; while the absolute F8WT and mFSST scores improved between timepoints, individuals’ relative standing did not change (all *r* > 0.91). Therefore, ICC analyses were deemed appropriate for evaluating reliability in this subset.

Table 2 presents between-days, test–retest reliability results, SEMs, and MDCs for the sample. ICC point estimates (ICC_3,k_) surpassed 0.90 for average gait speed over 3 trials as evaluated with the 10mWT; average F8WT time and number of steps obtained from 2 trials; and average 360TT and mFSST times, obtained from 4 and 2 trials, respectively. ICC point estimates and 95%CIs were ≥ 0.70 for average number of steps for the 360TT (ICC_3,k_ = 0.85; 95%CI: 0.70-0.92) and best time on the FSST (ICC_3,1_ = 0.87; 95%CI: 0.71-0.94). MDC_90_ values were 10 to 33% of sample means, while MDC_95_ values were 13 to 39% of sample means, with the largest MDCs for average number of steps during the 360TT and time to complete the mFSST, regardless of ‘average’ or ‘best’ performance. Lower MDCs for average mFSST performance support using the average of 2 trials, when available, but 6 participants, i.e., 16% of the sample, had at least 1 invalid trial, suggesting averaging might not be possible in all ambulatory individuals with AMC.

**Table 2:** Between-Days, Test–Retest Reliability Results

Outcome Measure	1^st^ Testing Session Mean ± SD	2^nd^ Testing Session Mean ± SD	ICC (95%CI)	SEM	MDC_90_	MDC_95_
10mWT, m/sec (*n* = 38)	1.02 ± 0.20	1.01 ± 0.21	.95 (.90, .97)	0.05	0.11	0.13
F8WT, sec (*n* = 37)^×^	7.77 ± 2.27	7.51 ± 2.01	.96 (.92, .98)	0.43	0.99	1.19
F8WT, steps (*n* = 37)	13.9 ± 3.1	13.7 ± 2.7	.91 (.82, .95)	0.87	2.02	2.42
360TT, sec (*n* = 38)	2.80 ± 1.03	2.84 ± 0.99	.97 (.93, .98)	0.17	0.41	0.48
360TT, steps (*n* = 38)	6.2 ± 1.4	6.2 ± 1.6	.85 (.70, .92)	0.60	1.39	1.66
mFSST-average, sec (*n* = 32)^β^	11.81 ± 5.21	10.52 ± 4.25	.94 (.80, .98)	1.16	2.70	3.23
mFSST-best, sec (*n* = 38)	12.01 ± 4.75	10.90 ± 4.15	.87 (.71, .94)	1.61	3.73	4.46

^x^*n* = 1 missing trial during 1^st^ testing session

^β^*n* = 8 missing trials among 6 participants

*Abbreviations*: *ICC* Intraclass Correlation Coefficient, *CI* Confidence Interval, *SEM* Standard Error of Measurement, *MDC* Minimal Detectable Change, *10mWT* 10-m Walk Test, *F8WT* Figure-of-8 Walk Test, *360TT* 360-degree Turn Test, *mFSST* modified Four Square Step Test, *m* meters, *sec* seconds

## Discussion

Outcome measures enable objective evaluation of functional changes and can inform clinical decisions, predict future ability, and fulfill healthcare documentation requirements [48]. Kennedy and colleagues called for core gait and functional ambulation outcome measures for use in pediatric clinical and research settings [16]. This study is a first step towards a possible core set of performance-based functional outcome measures for use in adolescents and adults born with AMC. Good-to-excellent between-days, test–retest reliability was found for the 10mWT, F8WT, 360TT, and mFSST, which are currently used in other patient populations. ICCs ≥ 0.90 suggest gait speed per the 10mWT, as well as evaluation of curved path walking and dynamic balance per the timed F8WT and 360TT, may be reliably evaluated among individuals with AMC on two separate occasions. Provided MDCs may enable clinicians to determine whether changes surpass measurement error and indicate ‘true change’ in their patients with AMC, i.e., pre-to-post rehabilitation (using MDC_90_) and pre-to-post surgery (using MDC_95_). Additionally, based on ICCs ≥ 0.70, evaluation of quality of movement, i.e., number of steps, as well as dynamic balance per the mFSST may have clinical trials utility in evaluating changes among groups of ambulatory individuals with AMC, although a potential floor effect should be considered when using the average of 2 mFSST trials, due to ‘invalid’ trials.

While our study did not include a control group, it appears our individuals with AMC are presenting with worse gait speed and dynamic balance when compared to controls and peers with other lower-extremity pathologies. Among typically-developing children and young adults, ‘self-selected’ gait speeds for 11–30 year olds are, on average, 1.28–1.36 m/sec, [49] which is significantly faster than speeds obtained in our participants with AMC (i.e., 1.01–1.02 m/sec). Further, Scott et al. found gait speeds of 1.2 ± 0.2 m/sec among adolescents and young adults with hip dysplasia (*n* = 24), suggesting gait speeds with AMC, where multiple lower-limb regions are typically involved, are worse [23]. Collectively, results highlight the importance of evaluating and addressing reduced gait speed among individuals with AMC, particularly since ‘self-selected’ gait speed is better correlated to perceived gait quality when compared to other performance-based measures, like the 6-Minute Walk Test, in young adults with congenital, mobility-limiting conditions [50]. Scott et al. also reported adolescents and young adults with hip dysplasia (*n* = 24) had FSST times of 6.6 ± 2.5 s, as compared to controls (*n* = 21; 4.0 ± 0.7 s) [23]. Individuals with AMC in our study had mFSST times that were double that of individuals with single-joint involvement and triple that of controls. Hence, with AMC, dynamic balance appears considerably compromised.

Mean 360TT times among young healthy adults (*n* = 34) have been reported to be 2.2 s, [35] which is about 20% faster than timed 360TT among our participants with AMC. Among children and young adults who are typically developing, peak turn velocity during 180 degree turns are, on average, 221–289 degrees/sec [49]. Among our participants with AMC, as evaluated with a ‘quick’ 360-degree turn, mean turn velocity was about 129 degrees/sec. Combined, data suggests impaired turning with AMC. As community-ambulation requires frequent turning, [31] and impaired turning has been associated with recurrent falls, [32] it may be imperative to incorporate turning into gait training among individuals with AMC.

Our participants with AMC had better FSST performance (median: 10.52–12.01 s) than children, aged 5–12 years, with Down syndrome and cerebral palsy, (i.e., mean: 18.7 ± 5.7 s), [40] which might be due to use of the mFSST without canes in our study and/or impaired cognition or inattention in the aforementioned pediatric study. Conversely, our participants had worse dynamic balance performance as compared to adults with unilateral lower-limb amputation [51] despite being younger and our use of the mFSST; [39] differences might be attributed to multi-region, lower-limb involvement with AMC.

Our between-days, test–retest reliability findings for performance-based tests are generally similar to reports among other patient populations [23, 24, 28, 29, 34–37, 39, 40, 51, 52]. For example, among adolescents and young adults with hip dysplasia and controls (*n* = 34), Scott et al. reported self-selected gait speed test–retest reliability over 10 timed meters of a 14-m course (ICC_2,1_ = 0.93; 95%CI: 0.87-0.96), [23] similar to our 10mWT reliability results (ICC_3,k_ = 0.95; 95%CI: 0.90-0.97). Among children with neurological conditions, Graser et al. also reported similar between-days reliability using 10-timed meters over a 14-m course (ICC_2,1_ = 0.90; 95%CI: 0.80-0.95). [24] A lower MDC_95_, i.e., 0.13 m/s in our study and 0.18 m/sec in another study, [52] as compared to 0.35 m/sec in the Scott et al. study, [23] may be due to trial averaging.

Between-days test–retest reliability for the F8WT, performed at a given individual’s self-selected speed, has been reported by Hess et al. for older adults (*n* = 18; time: ICC = 0.84; 95%CI: 0.62-0.94; number of steps: ICC = 0.82; 95%CI: 0.59-0.93) [26] and among individuals post-stroke (*n* = 35; time: ICC_2,1_ = 0.98; 95%CI: 0.96-0.99) [28]. Better F8WT reliability among our participants with AMC (time: ICC_3,k_ = 0.96; 95%CI: 0.92-0.98; steps: ICC = 0.91; 95%CI: 0.82-0.95) compared to the Hess et al. study [26] may be secondary to our larger sample size and averaging two trials. For F8WT at fast speed using 2 loops, among older women, Jarnlo and Nordell reported test–retest reliability comparable to our study (*n* = 30; ICC_3,1_ = 0.93; 95%CI: 0.85-0.97) [29]. To our knowledge, comparative MDC values are unavailable.

The timed 360TT has published between-days test–retest reliability among individuals with Multiple Sclerosis (*n* = 61; ICC_2,2_ = 0.91-0.96; 95%CI: 0.86-0.97; MDC_95_ = 1.5 s), [35]. Parkinson’s Disease (*n* = 14; ICC = 0.80; lower bound of 95%CI: 0.66), [34] and post-stroke (*n* = 37; ICC_3,2_ = 0.82-0.95; 95%CI: 0.66-0.98; MDC_95_ = 0.8–1.2 s) [36]. Between-days test–retest reliability for number of steps is reported in Parkinson’s Disease (*n* = 14; ICC = 0.77, lower bound of 95%CI: 0.61) (34) and among older adults (ICC = 0.92) [37]. We report similarly good-to-excellent reliability for 360TT time (ICC_3,k_ = 0.97, 95%CI: 0.93-0.98) and number of steps (ICC_3,k_ = 0.85; 95%CI: 0.70-0.92), but a lower MDC_95_ for 360TT time (i.e., 0.5 s), suggesting changes in turning speed may be more easily identified among individuals with AMC as compared those with other neurological conditions.

While many studies report FSST or mFSST *within-day* test–retest reliability, only a few report *between-days* test–retest reliability, [23, 39, 40, 51] which better parallels ‘evaluations’ and ‘re-evaluations’ in clinical practice, upon which patient improvements are determined. Among children with neurological conditions (*n* = 30), FSST between-days, test–retest reliability (ICC_1,1_ = 0.54-0.89; 95%CI: 0.24-0.95) is reported [40]. Among adolescents and young adults with hip dysplasia and controls (*n* = 34) and adults post-stroke (*n* = 17), between-days, test–retest reliability for average FSST performance (ICC_2,1_ = 0.93; 95%CI: 0.87-0.96; MDC_95_ = 1.66 s) and best mFSST performance (ICC_3,1_ = 0.90; 95%CI: 0.68-0.97) are reported [23, 39]. Between-days, test–retest reliability for best FSST performance is also reported among adults with unilateral lower-limb amputation (*n* = 60; ICC_2,1_ = 0.97; 95%CI: 0.94-0.98; MDC_90_ = 2.0 s) [51]. Our mFSST reliability (ICC = 0.87-0.94; 95%CI: 0.71-0.98) was comparable to aforementioned adult studies; (23, 39, 51). MDCs, i.e., 2.70–4.46 s, were less than those reported among children with neurological conditions, i.e., 5.29 s. [40].

## Study strengths and limitations

Our gait speed assessments were scientifically robust as we used a static starting position for all trials, allowed 2.0 m for acceleration, and provided standardized examiner instructions (‘usual pace’); failure to control for any of these factors may negatively impact test–retest reliability. [53] Nevertheless, we could not establish the minimal clinically important difference (MCID) for gait speed, or the other performance-based measures, given the study design. Based on a systematic review of gait speed among individuals with pathology, however, the MCID is likely around 0.1 m/s, [54] which is similar to the MDCs calculated in our study.

Study strengths include recruitment of a mixed sample of adolescents and adults with AMC, as well as selection of clinically-feasible outcome measures, which may enhance clinician adoption [55]. But, we acknowledge some additional limitations. First, without access to medical records, we could not confirm self-reported data, including whether participants were amyoplasia- or distal-type arthrogryposis, as defined by Hall et al [5]. The extent of limb involvement, however, would suggest the majority of our participants might be classified within the amyoplasia-type subgroup. Second, we standardized testing based on current practice, where individuals complete 1–3 recorded trials; we did not evaluate for practice effects (by completing trials until fatigue), which might have resulted in underestimation of performance. Third, we did not specifically target a care-seeking sample, who might have had worse mobility status or greater between-days pain fluctuations, which might have increased floor effects for some measures or negatively influenced test–retest reliability. Finally, performance in a laboratory, or clinical setting, may not reflect real-world performance; for example, gait speed among minors with developmental disorders has been reported to be slower in the real-world when compared to laboratory-obtained gait speed [56].

## Conclusions

This study supports subsequent research evaluating linear and curved-path walking, as well as dynamic balance, via the 10mWT, F8WT, 360TT, and mFSST, among individuals with AMC. Future studies with larger sample sizes may seek to establish MCIDs, evaluate floor and ceiling effects among a more diverse sample in terms of mobility and musculoskeletal pain, and determine responsiveness of these performance-based measures to commonly employed interventions, such as bracing, rehabilitation, and surgery, in this patient population. In the interim, practitioners may adopt the 10mWT, F8WT, and 360TT and use provided MDCs when evaluating individuals with AMC for objectively determining intervention effectiveness.

## Data Availability

The dataset generated and analyzed during the current study is not publicly available due the prevalence of arthrogryposis multiplex congenita and the limited geographic regions where data collections occurred, resulting in the potential for individual participants to be identified. The dataset is, however, available from the corresponding author on reasonable request at megsions@udel.edu through use of data sharing agreement that takes additional steps to protect participant confidentiality.

## Abbreviations

AMC: Arthrogryposis Multiplex Congenita
F8WT: Figure-of-8 Walk Test
FSST: Four Square Step Test
GFAQ: Gillette Functional Assessment Questionnaire
ICC: Intraclass correlation coefficients
MCID: Minimal clinically important difference
MDC: Minimal detectable change
MDC_90_: Minimal detectable change at 90% confidence level
MDC_95_: Minimal detectable change at 95% confidence level
mFSST: Modified Four Square Step Test
SEM: Standard error of measurement
10mWT: 10-m Walk Test
360TT: 360-Degree Turn Test
